# Hadron therapy information sharing prototype

**DOI:** 10.1093/jrr/rrt037

**Published:** 2013-07

**Authors:** Faustin Laurentiu Roman, Daniel Abler, Vassiliki Kanellopoulos, Gabriel Amoros, Jim Davies, Manjit Dosanjh, Raj Jena, Norman Kirkby, Ken Peach, Jose Salt

**Affiliations:** 1 CERN, European Organization for Nuclear Research, CH-1211 Geneva 23, Switzerland; 2 IFIC, Instituto de Física Corpuscular, Apartado de Correos 22085, E-46071 Valencia, Spain; 3 Present address: EBG MedAustron GmbH, Marie Curie-Strasse 5, A-2700 Wiener Neustadt, Austria; 4 Department of Computer Science, University of Oxford, Wolfson Building, Parks Road, Oxford OX1 3QD, UK; 5 Faculty of Engineering & Physical Sciences, University of Surrey, Guildford, Surrey GU2 7XH, UK; 6 PTCRi, Particle Therapy Cancer Research Institute, Denys Wilkinson Building, Keble Road, Oxford OX1 3RH, UK; 7 Cambridge University Hospitals NHS Foundation Trust, Hills Road, Cambridge, CB2 0QQ, UK

**Keywords:** hadron therapy, proton therapy, data federation, web portal, eHealth, cancer informatics

## Abstract

The European PARTNER project developed a prototypical system for sharing hadron therapy data. This system allows doctors and patients to record and report treatment-related events during and after hadron therapy. It presents doctors and statisticians with an integrated view of adverse events across institutions, using open-source components for data federation, semantics, and analysis. There is a particular emphasis upon semantic consistency, achieved through intelligent, annotated form designs. The system as presented is ready for use in a clinical setting, and amenable to further customization. The essential contribution of the work reported here lies in the novel data integration and reporting methods, as well as the approach to software sustainability achieved through the use of community-supported open-source components.

## INTRODUCTION

There is a need for increased data sharing in cancer care and cancer research [1, 2]. In the case of hadron therapy, this need is particularly pronounced. Also known as particle or ion beam therapy, it involves the innovative use of protons and carbon ions [3], and while this promises significant advantages, more information is required to support adoption, to determine suitability, and to support treatment planning. Furthermore, there are relatively few treatment centres, and patients often cross national boundaries for treatment. There is a requirement for effective follow-up to compare outcomes and establish efficacy [4], and a European initiative has been established to provide this [5, 6].

An analysis of the hadron therapy domain, considering the requirement for data interoperability and the achievements of existing eHealth initiatives, has informed the design of a ‘Hadron therapy Information Sharing Prototype’ (HISP), as a gateway to patient information held in multiple hospital databases and a means of supporting patient follow-up in multi-centre clinical studies [7, 8].

To demonstrate the functionality of the system, we focus on an adverse-event-reporting scenario—a key component of comprehensive patient follow-up. The reporting of adverse, treatment-related events is part of the patient management process, beginning with the initial visit where a baseline is assessed, continuing during treatment with each clinical review, and then at intervals during follow-up. This demonstration covers the main aspects of system functionality: patients and doctors report an adverse event as structured, coded information; this information is integrated into medical records at treatment centres, and is then available, across a distributed architecture, under role-based access control.

The prototype was developed within the PARTNER Project, as a collaborative effort between three Marie-Curie Early Stage Researchers and four institutions: CERN, IFIC, the University of Oxford, and the University of Surrey [5]. This paper reports upon the design of the architecture and principal components, as well as upon the benefits and limitations of the existing implementation.

## MATERIALS AND METHODS

The system provides a single access point for patient information distributed across multiple hospital databases. At the design level, it can be seen as a generic server infrastructure, with services for data integration and presentation, and other services for reporting and analysis.

### System architecture

The particular challenges within this domain include (i) a lack of interoperability between existing clinical care systems; (ii) the distribution of information pertaining to an individual patient across multiple systems; and (iii) a lack of consistent and amenable policies for data access. These challenges had a profound influence upon the system design, the data model, and the development process.

**Fig. 1.**
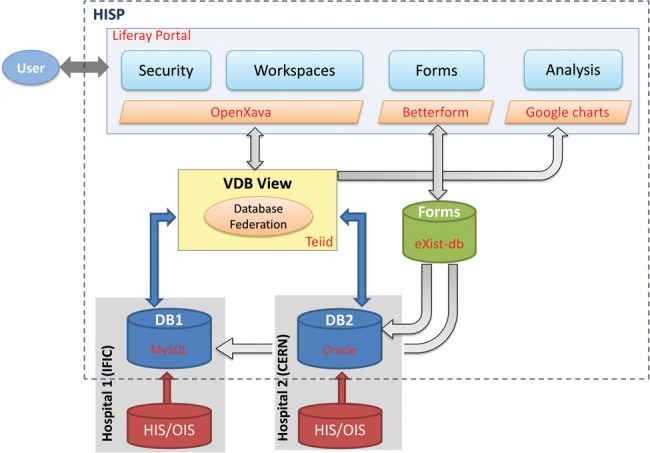
HISP architecture: data stored in DB1 and DB2 is held locally but available remotely.

The architectural design was based upon the storyline or pathway of an individual patient undergoing hadron therapy [9]. In this storyline, data is collected in two countries, from different actors: doctors and patient. Data is held within local networks, but made available to external services. The prototypical infrastructure was realized on four servers distributed across two sites (Fig. 1) [10, 11]:

- CERN server 1: presentation services, user interface
- CERN server 2: integration services
- CERN server 3: metadata and reporting services, data capture
- IFIC server 4: database located at second site.

### Databases

The hospital data repositories, Hospital or Oncology Information Systems (HIS/OIS), export data to local HISP databases remaining under local governance (DB1 and DB2). The basic data model adopted for this scenario represents a ‘care summary’ view of the data, suitable for use across institutions. Raw images, laboratory reports, and treatment plans are not shared in this scenario. Sample data to validate the system was generated from published clinical trials, and imported into the hospital databases for testing purposes.

The model [12] comprises four tables of data: patient information (e.g. demographics, history); tumour information (e.g. type, staging); treatment information (e.g. dose and beam quality); and adverse-events information (e.g. scoring system, adverse event, grade, time of onset). The data in the first three tables would be imported automatically from hospital systems; only the adverse-events data needs to be acquired separately. The structure of the model allows for the submission of patient- and doctor-reported event information to different data standards. In the example schema, however, any reported event has to be represented as a combination of ‘adverse-event name’, ‘severity score’ and ‘scoring system’. For the inclusion of patient-reported outcome measures, this would require an initial transformation step in the reporting services, anticipated in the architecture design.

One part of the generated patient data was stored in the IFIC ‘MySQL’ server (DB1); the other was stored in a CERN ‘Oracle’ database instance (DB2) [13, 14]. As ‘Oracle’ databases, often used in hospital environments, have different characteristics to ‘MySQL’ databases (operations, schema and access), this allowed us to test low-level interoperability and integration functions.

### Data integration

The architecture relies upon ‘data federation’ to provide an integrated view across heterogeneous data sources, presenting their aggregation as a virtual database. This virtual database does not contain the data itself, but instead holds information about the data locations, types, and access procedures. The data remains within the source organisations, which are able to determine the extent to which it may be accessed across the system.

‘JBoss Teiid’ was used as the federation platform [15], hosted on CERN server 2, and used to integrate the two databases, ‘MySQL’ and ‘Oracle’, to produce a virtual database (VDB) view. The view was restricted to address the particular requirements of the adverse-event-reporting scenario [10].

### Portal

The user interface for the system was based upon the ‘Liferay portal’, an enterprise-level, open-source standards-compliant web platform [16]. The medical domain has particular requirements for standards, certification, and support; this technology addressed these, while providing a range of useful, packaged functionality. For the interface, we used ‘Openxava’: a ‘Java’-based framework for the rapid development of internet applications [17], compatible with ‘Liferay’. The combined framework allowed us to produce interfaces that were both easy to use and easy to develop.

### Security

Three primary roles are defined for authorization (doctor, patient, researcher), together with two further, secondary roles (new user and administrator) [10]. ‘Doctor’ is the most privileged role, affording access to all patient data within the federation. ‘Researcher’ allows a simple statistical analysis. ‘Patient’ affords access to the follow-up forms. A given user can be assigned multiple roles, depending upon the context of current activity.

Communication with the HISP portal (server) is secured using the Transport Layer Security (TLS) version 1.2 encryption protocol. Users can login via password-based authentication or public key certificates loaded into the client browser: the second approach, suitable for use within a controlled hospital environment, facilitates access and provides increased security overall [18].

### Data reporting

The data reporting functionality was designed for web-based structured reporting of medical findings, with minimum standards of documentation, facilitating the re-use of data across contexts.

### Metadata services

To facilitate re-use, the intended meaning of the data must be documented and be available as ‘metadata’ in a computable form. Metadata registries serve as common reference points for metadata standards, and provide services for metadata registration, curation and versioning. ISO/IEC 11179 is a standard for metadata registries, addressing ‘the semantics of data, the representation of data, and the registration of the descriptions of that data’ to promote common understanding, harmonization, and re-use [19]. A key part of this standard is the notion of a common data element, a fundamental unit of metadata that may be applied to data collected or managed in a different context. A data element is intended to provide a full description of the meaning of a given observation. To provide the required metadata support for the adverse-event scenario, we used the ‘CancerGrid’ metadata registry (cgMDR) [20], an open-source implementation of the ISO/IEC 11179 standard.

### Data reporting services

The reporting services had to be widely accessible: to provide verification of data entry prior to submission, and to allow for changes in requirements to be easily accommodated through the versioning of data-capture instruments and data schemas. This latter aspect is particularly important for early-phase clinical studies, where the set of observations to be made is not finalized at the outset of the study. It is important also in the context of new or evolving reporting instruments and standards, e.g. where new measures are being devised for the reporting of ‘subjective’ adverse events after therapy.

The ‘Extensible Markup Language’ (XML) [21] was chosen as the serialization format for submitted data, reflecting a flexible document-centric approach in which every form submission corresponds to a single observation record. The validity of any submitted record can be verified using an XML schema. By annotating the schema files with SAWSDL references [22] pointing to the respective data elements registered in the metadata registry, collected data is prepared for future data-integration scenarios.

Data-reporting forms were developed in ‘XForms’ [23], an XML-based standard recommended by the World Wide Web Consortium (W3C) for the declarative definition of web forms. We used ‘betterFORMS’, a server-side implementation of XForms [24], to make the forms accessible using standard web technologies. Validation rules within the form specification are used to check data prior to storage in a native XML database: ‘eXist-db’ [25], was chosen for this purpose.

The identity of the submitter (patient or doctor and respective credentials), the subject (patient and credentials), and the associated treatment centre is verified at form submission. A user-defined extract of the reported data, possibly involving data transformations to comply with the target schema, is then transmitted into the local database (DB1 or DB2 in Fig. 1) and becomes available to federated queries. In the prototype implementation, this functionality is provided by a ‘ModPython’ [26] script, retrieving the submitted XML record, performing the necessary data transformations, and generating the query statements to insert the data into the database.

All of the above services are hosted on CERN server 3. Data-reporting forms were created for patient-reported outcome measures, based on existing questionnaires [27]. Forms were created for the submission of adverse-event reports, based on reporting standards such as CTCAE, RTOG and SomaLent [28–31].

### Analysis service

The federated data can be used to provide a range of analysis services. Depending upon the user's role, these can be customized to address specific needs. We used PHP and the Google charts API [32] to build a simple proof-of-concept report. This queries the VDB anonymously to produce statistical information about the cumulated adverse events: for example, a piechart display of the total numbers of the five most common adverse events. A range of parameters for the queries can be selected using drop-down menus.

## RESULTS

The HISP PARTNER prototype system demonstrates a particular approach to the acquisition and re-use of relevant medical data. The following features are seen as benefits or advantages of the approach:

- the ability to query data across a federation of heterogeneous database systems
- the availability of an easy-to-use portal interface with role-based access control
- the use of semantic web and metadata technologies to facilitate semantic annotation of data
- the support for versioning of patient-reported outcome and objective adverse-event measures.

## DISCUSSION

Although existing solutions for clinical data management—such as OpenClinica [33], caAERS [34], or the particle database system developed within the European ULICE-framework [35]—provide functionalities that the HISP prototype does not support, the system serves well to demonstrate a range of different and important features.

Rather than employing a traditional ‘data warehousing’ approach [35], HISP relies on ‘data federation’ to provide an integrated view across heterogeneous data sources. A warehousing approach requires a fixed model, less amenable to change, and could lead to concerns over data control and privacy. This was a particularly important consideration given the evolving nature of the science and medical practice, as well as the concrete specification of governance constraints upon medical data (typically, ‘the data stays within the hospital; only suitably abstracted versions of the data may be made available externally, and then only for clearly specified purposes’).

Where reporting scenarios involve multiple institutions, and where data collection may take place over a period of years, clear documentation of reporting intent is needed for subsequent, correct interpretation and re-use. As the scale of data collection increases, the importance of machine-readable documentation—computable metadata—increases also. The advantages of semantic annotation based upon metadata standards have also been demonstrated in [36]. An important feature of the HISP design is that annotation is part of the form design process, ensuring that data is associated, automatically, with a computable representation of its meaning at the point of collection.

A model-driven approach is used to automate the process of form implementation, minimizing the development effort involved [37], improving quality, and reducing the cost of validation [38]. Other reporting systems frequently provide the means of facilitating the form design process by providing ‘form builders’: some of these are simplistic [39]; others treat each form as an indivisible whole [33, 40], preventing comparison and re-use at a data-element or question level. As different conditions, different questions, and even different individuals may be presented with different forms [41], the ability to compare designs at a question level is essential for data re-use and integration. The ability to re-use designs at this level is equally essential for the harmonization of reporting standards and procedures. Lessons learned from the prototype development have informed the design of comprehensive metamodel, or domain-specific language, for form specification [42].

The use of open-source components is incidental to the design of the system, but makes an important contribution to its extensibility and potential for adoption. The purpose of the prototype is to establish technical feasibility, and significant extension would be required for wider deployment outside the context of the originating project: for example, a collection of standard interfaces to hospital databases and messaging systems. However, the system as it stands serves as a complete demonstration for the proposed approach, as well as a platform for further development.

## FUNDING

This work was supported by a Marie Curie Initial Training Fellowship of the European Community's Seventh Framework Programme under contract number [PITN-GA-2008-215840-PARTNER].
